# Synthesis, crystal structure and Hirshfeld surface analysis of 5,5-diphenyl-2-[2-(propan-2-yl­idene)hydrazin-1-yl]-4,5-di­hydro-1*H*-imidazol-4-one *N*,*N*-di­methyl­formamide hemisolvate

**DOI:** 10.1107/S2056989025000076

**Published:** 2025-01-10

**Authors:** Abderrazzak El Moutaouakil Ala Allah, Benson M. Kariuki, Walid Guerrab, Abdulsalam Alsubari, Musa A. Said, Joel T. Mague, Youssef Ramli

**Affiliations:** a Laboratory of Medicinal Chemistry, Drug Sciences Research Center, Faculty of Medicine and Pharmacy, Mohammed V University, Rabat, Morocco; b School of Chemistry, Cardiff University, Main Building, Park Place, Cardiff, CF10 3AT, United Kingdom; c Laboratory of Medicinal Chemistry, Faculty of Clinical Pharmacy, 21 September University, Yemen; d Department of Chemistry Faculty of Science Islamic University of Madinah, Madinah 42351 Saudi Arabia; e Department of Chemistry, Tulane University, New Orleans, LA 70118, USA; Venezuelan Institute of Scientific Research, Venezuela

**Keywords:** crystal structure, hydrogen bond, di­hydro­imidazolone, C—H⋯π (ring) inter­action

## Abstract

The asymmetric unit of the title structure, 2C_18_H_18_N_4_O_2_·C_3_H_7_NO, consists of two independent mol­ecules of the substituted imidazolone having different conformations, and one mol­ecule of solvent DMF. The two imidazolone mol­ecules are linked by N—H⋯N and C—H⋯O hydrogen bonds and the DMF is joined to one of these by an N—H⋯O hydrogen bond. Additional N—H⋯N and C—H⋯O hydrogen bonds link these groups into corrugated layers parallel to the (101) plane with the layers joined by C—H⋯π (ring) inter­actions. A Hirshfeld surface analysis indicated that H⋯H inter­actions account for over half of the inter­molecular contacts.

## Abstract 2

The asymmetric unit consists of two independent mol­ecules of the substituted imidazolone having different conformations, and one mol­ecule of solvent DMF. The two imidazolone mol­ecules are linked by N—H⋯N and C—H⋯O hydrogen bonds and the DMF is joined to one of these by a N—H⋯O hydrogen bond. Additional N—H⋯N and C—H⋯O hydrogen bonds link these groups into corrugated layers parallel to the (101) plane with the layers joined by C—H⋯π (ring) inter­actions.

## 1. Chemical context

In recent years, hydrazide-hydrazone derivatives have attracted increasing inter­est because of their wide range of applications in medicinal chemistry. In particular, the presence of an azomethine moiety (–NHN=CH–) in these compounds is often associated with their biological activity. *In vitro* studies on the toxicity of isoniazid on different cell lines have demonstrated that isoniazid induces cytotoxicity through apoptosis, leading to significant disruption of the cell cycle in mammalian cells (Naveen Kumar *et al.*, 2014 ▸). Recently, several new hydrazine and hydrazone derivatives based on hydantoin have been synthesized (Attanasi *et al.*, 2011 ▸) and have shown remarkable biological activity, especially as anti­tumor agents (Guerrab *et al.*, 2023 ▸). Additionally, hydantoin is an important pharmacophore in medicinal chemistry because of its numerous biological applications, including as an anti­bacterial (El Moutaouakil Ala Allah *et al.*, 2024*a* ▸,*b* ▸), anti­epileptic (El Moutaouakil Ala Allah *et al.*, 2024*c* ▸), anti­plasmodial (Chin *et al.*, 2024 ▸), and anti­viral (Pardali *et al.*, 2023 ▸) agent. Continuing our research in this field, we synthesized the title compound 5,5-diphenyl-2-[2-(propan-2-yl­idene)hydrazin-1-yl]-4,5-di­hydro-1*H*-imidazol-4-one *N*,*N*-di­methyl­formamide hemisolvate *via* a condensation reaction between 2-hydrazinyl-4,4-diphenyl-1*H*-imidazol-5(4*H*)-one and acetone in the presence of acetic acid as a catalyst. We determined its mol­ecular and crystalline structures, and conducted a Hirshfeld surface analysis.
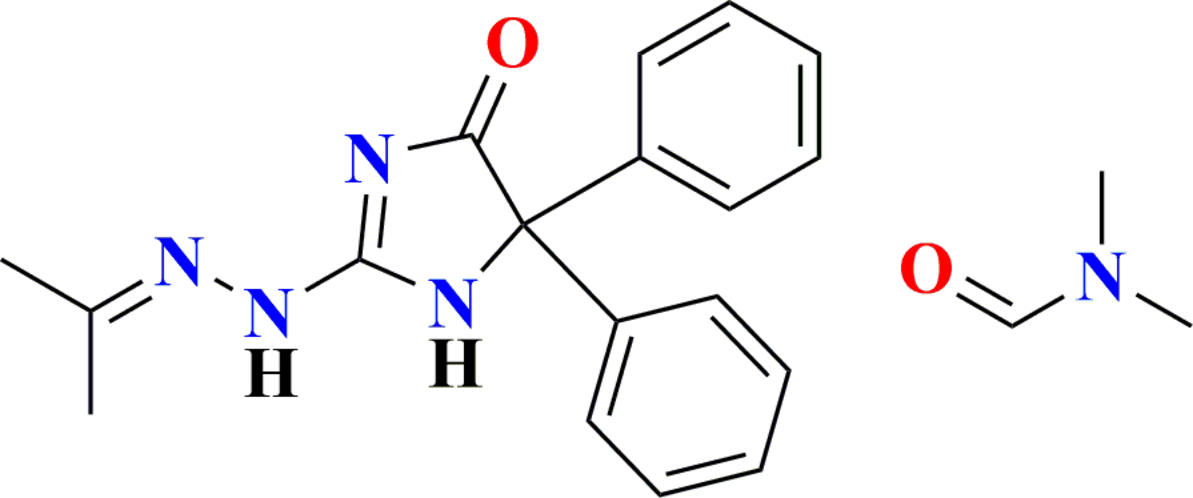


## 2. Structural commentary

**Figure 1:**
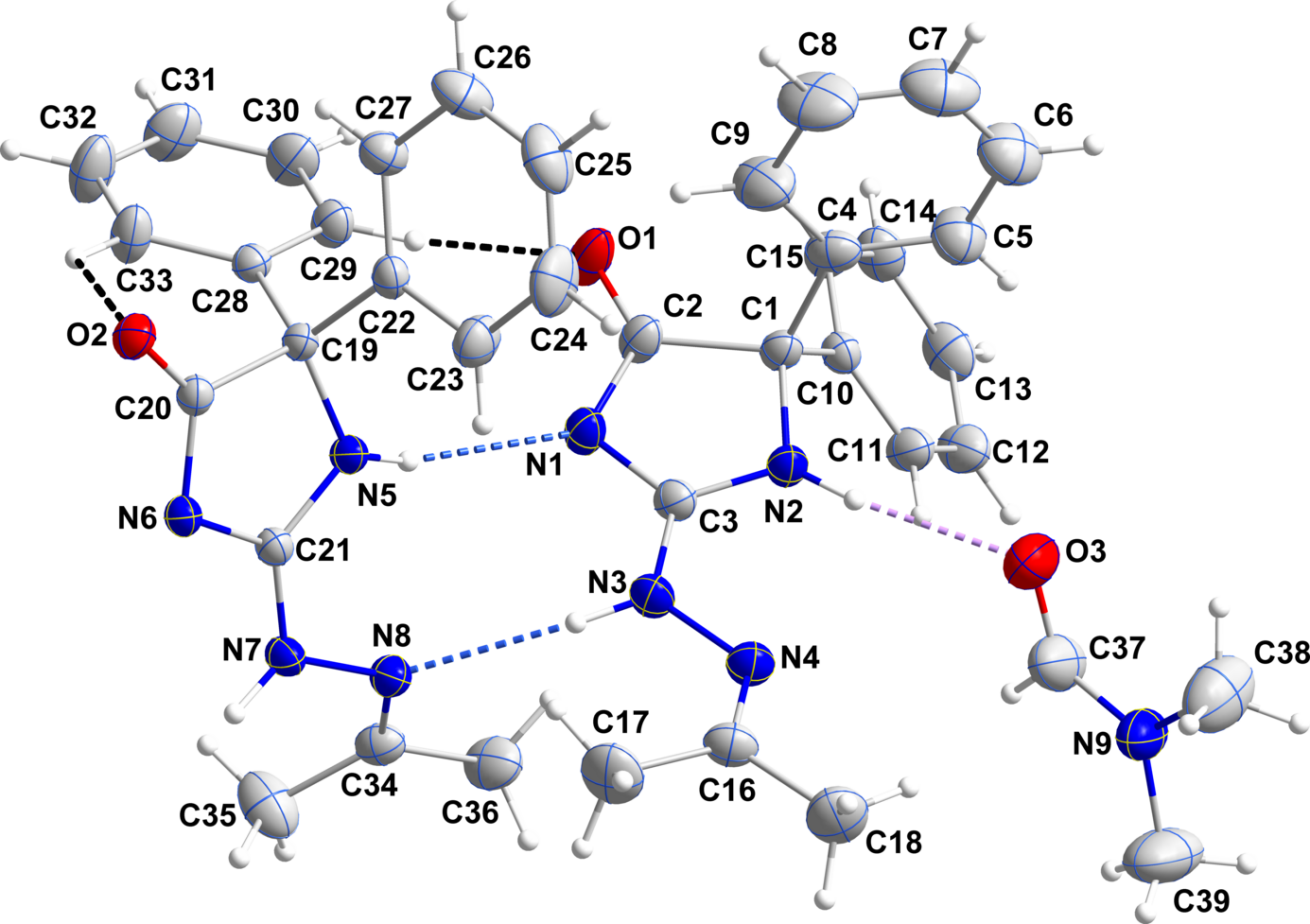
Perspective view of the asymmetric unit with numbering scheme and 30% probability ellipsoids. The N—H⋯N, N—H⋯O and C—H⋯O hydrogen bonds are depicted, respectively, by blue, violet and black dashed lines.

**Figure 2:**
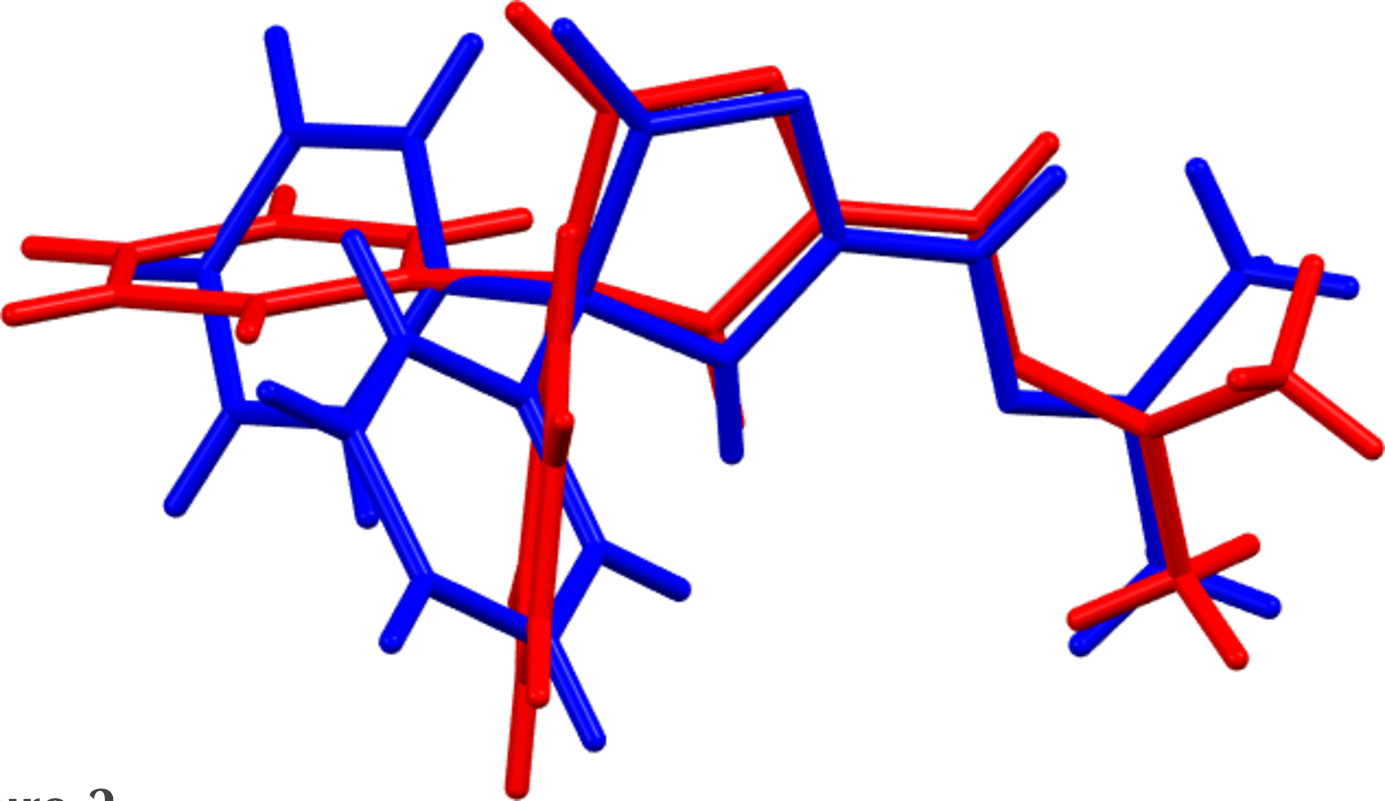
Overlay of the two independent mol­ecules (mol­ecule containing O1 in blue and that containing O2 in red).

The asymmetric unit consists of two independent mol­ecules of the imidazolone derivative and one mol­ecule of solvent di­methyl­formamide (DMF) (Fig. 1 ▸). The independent mol­ecules differ in the orientations of the phenyl rings and in the departure from planarity of the imidazolone rings (Fig. 2 ▸). For the mol­ecule containing O1, N2 is 0.0387 (12) Å to one side of the mean plane of the imidazolone ring (r.m.s. deviation of the fitted atoms = 0.0285 Å) while C1 is 0.0358 (12) Å to the other side. In that containing O2, N5 is 0.0520 (11) Å to one side of the mean plane of the imidazolone ring (r.m.s. deviation of the fitted atoms = 0.0399 Å) while C19 is 0.0519 (10) Å to the other side. In fact, the latter ring is sufficiently non-planar as to be amenable to a Cremer–Pople puckering analysis (Cremer & Pople, 1975 ▸), which gave *Q*(2) = 0.08193 (1) Å and φ(2) = 206.4 (12)° and a conformation having a twist on the N5—C19 bond. In the first mol­ecule, the mean planes of the C4–C9 and the C10–C15 rings are inclined to that of the imidazolone ring by 77.64 (9) and 55.80 (7)°, respectively, while the corresponding angles for the C22–C27 and the C28–C33 rings are 76.92 (8) and 70.98 (6)°, respectively. Another difference is in the conformation of the propanylidenehydrazineyl substituent where the N4—N3—C3—N1 and the N8—N7—C21—N6 torsion angles are 178.35 (18) and −174.16 (16)°, respectively. The sum of the angles about N3 and N7 are 358.2 (15) and 359.6 (14)°, respectively, indicating participation of their lone pairs in π bonding to adjacent atoms. This appears to be primarily with C3 and C21 as the N3—C3 and N7—C21 bond lengths are 1.332 (2) and 1.310 (2) Å, respectively.

## 3. Supra­molecular features

**Figure 3:**
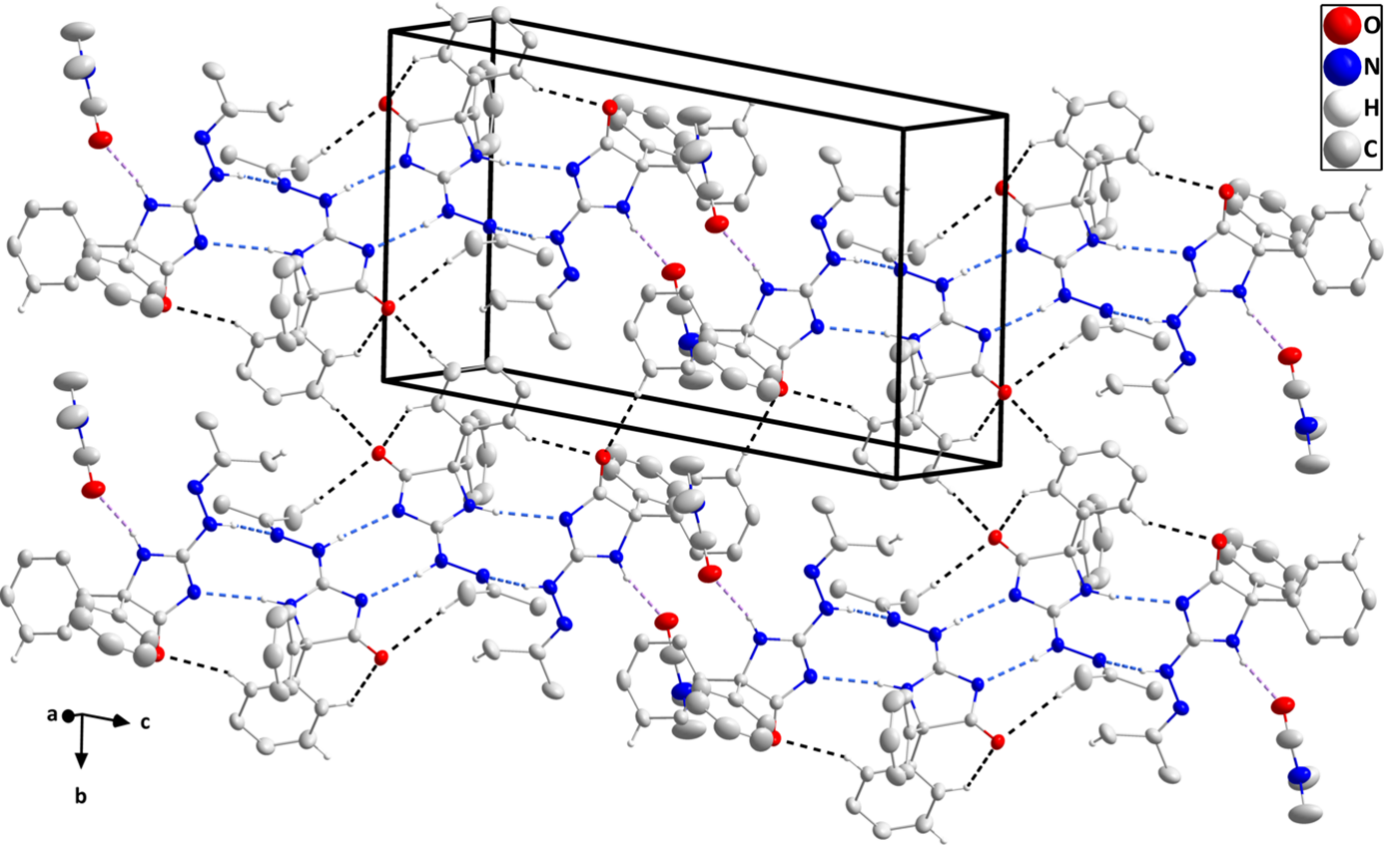
A portion of one layer projected onto the (101) plane with the N—H⋯N, N—H⋯O and C—H⋯O hydrogen bonds depicted, respectively, by blue, violet and black dashed lines. Hydrogen atoms not involved in these inter­actions are omitted for clarity.

**Figure 4:**
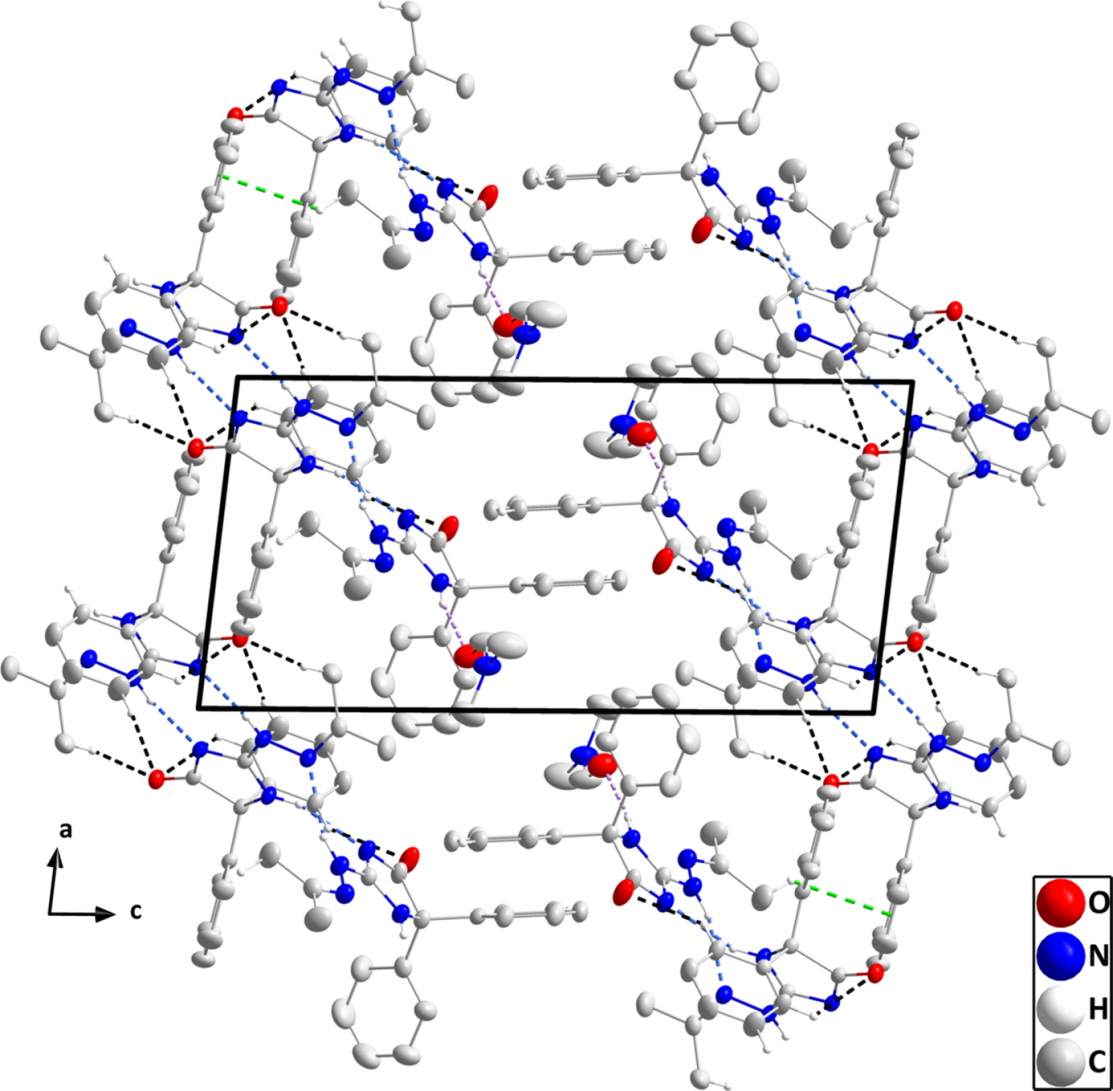
The packing viewed along the *b*-axis direction with N—H⋯N, N—H⋯O and C—H⋯O hydrogen bonds depicted, respectively, by blue, violet and black dashed lines. The C—H⋯π (ring) inter­actions are depicted by green dashed lines and hydrogen atoms not involved in these inter­actions are omitted for clarity.

The two independent mol­ecules are connected by N3—H3⋯N8, N5—H5*A*⋯N1 and C29—H29⋯O1 hydrogen bonds while the solvent DMF mol­ecule is attached by an N2—H2⋯O3 hydrogen bond (Table 1 ▸ and Fig. 1 ▸), thereby grouping the components of the asymmetric unit into the fundamental building block of the full crystal structure. N7—H7*A*⋯N6^i^ hydrogen bonds (Table 1 ▸) connect two such blocks into centrosymmetric dimers, which are then linked into chains parallel to the (101) plane by inversion-related C14—H14⋯O1^ii^ hydrogen bonds (Table 1 ▸). The chains are linked by inversion-related C32—H32⋯O2^iv^ hydrogen bonds into corrugated layers parallel to the (101) plane (Table 1 ▸ and Fig. 3 ▸). The layers are linked by C17—H17*B*⋯*Cg*5^iii^ inter­actions (Table 1 ▸ and Fig. 4 ▸), generating the full 3-D structure.

## 4. Database survey

**Figure 5:**
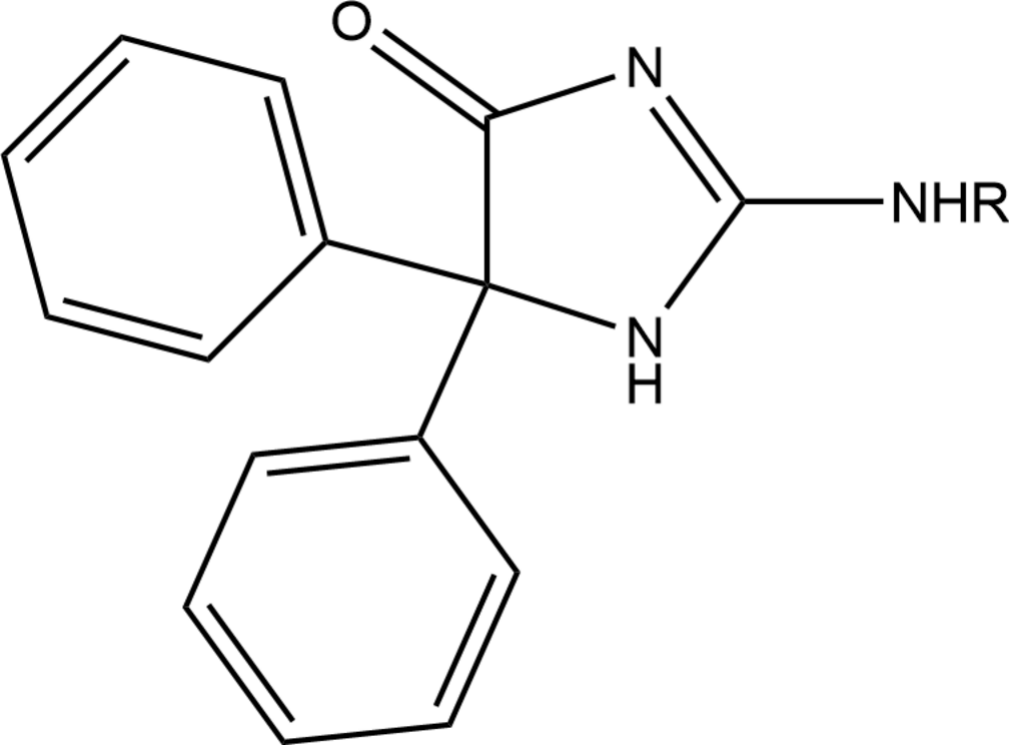
The fragment used in the database search.

A search of the Cambridge Structural Database (CSD, updated to June 2024; Groom *et al.*, 2016 ▸) with the fragment shown in Fig. 5 ▸ gave two hits, one with *R* = CH_2_COOEt (REFREB; Karolak-Wojciechowska *et al.*, 1998 ▸) and the other with *R* = 4-hy­droxy­phenyl (HOHBAL; El Moutaouakil *et al.*, 2024 ▸). Both structures have one mol­ecule per asymmetric unit and no solvent. In REFREB, the dihedral angles between the mean planes of the phenyl rings and that of the imidazolone ring are 63.3 (2) and 82.9 (2)° and the imidazolone ring has an ‘open envelope’ conformation. The exocyclic C—N bond length to the imidazolone ring is 1.325 (4) Å, suggesting involvement of the nitro­gen lone pair in N→C π bonding. The corresponding dihedral angles in HOHBAL are 73.33 (9) and 50.78 (11)°, which are very similar to those in one of the mol­ecules of the title compound. The imidazolone ring deviates from planarity by 0.021 (2) Å and the exocyclic C—N bond is 1.329 (3) Å, again indicating nitro­gen lone pair involvement in N→C π bonding.

## 5. Hirshfeld surface analysis

**Figure 6:**
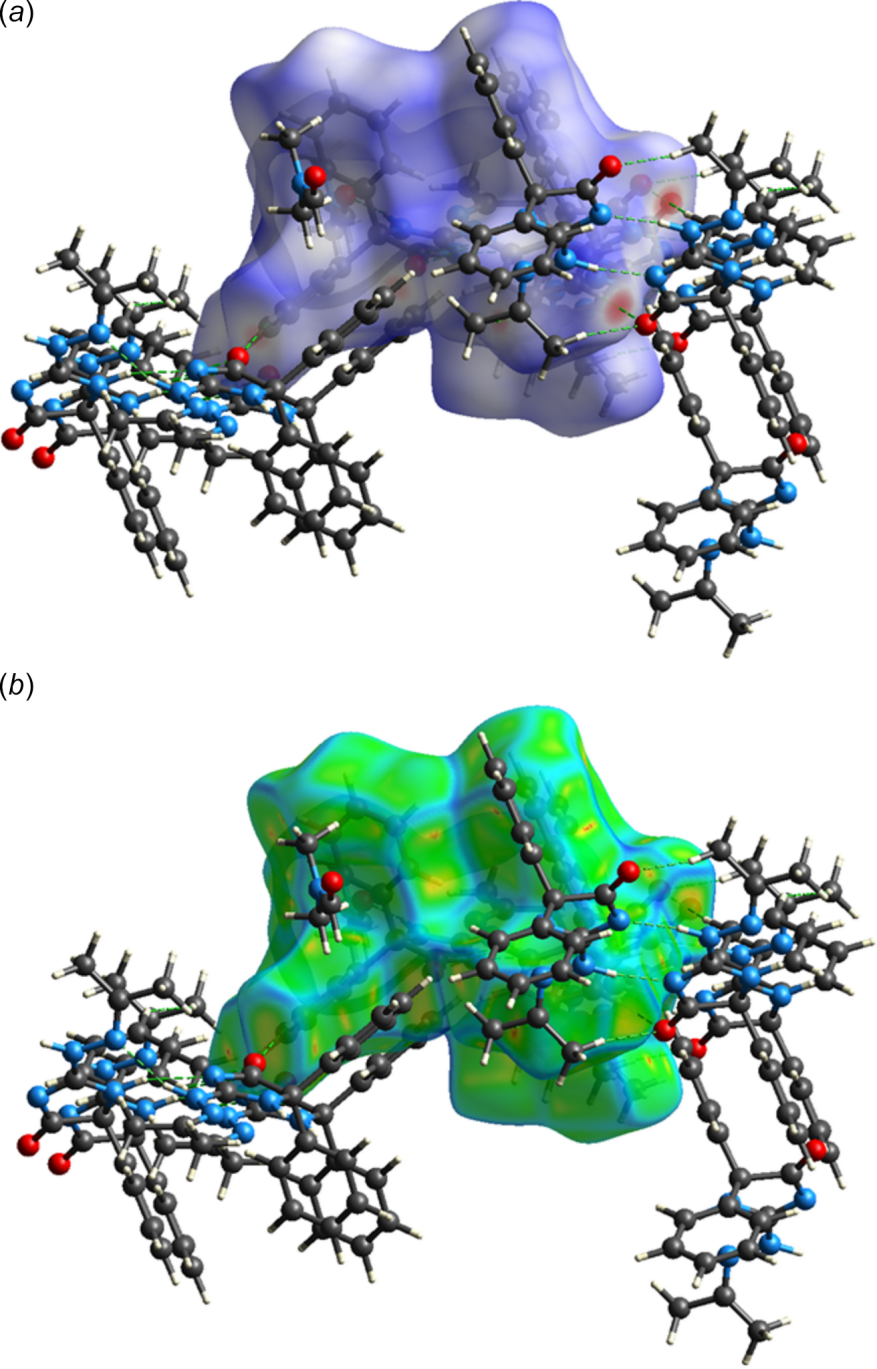
The Hirshfeld surface of the asymmetric unit with several neighboring mol­ecules plotted over (*a*) *d*_norm_ and (*b*) the curvature function. Hydrogen bonds are depicted as dashed lines.

**Figure 7:**
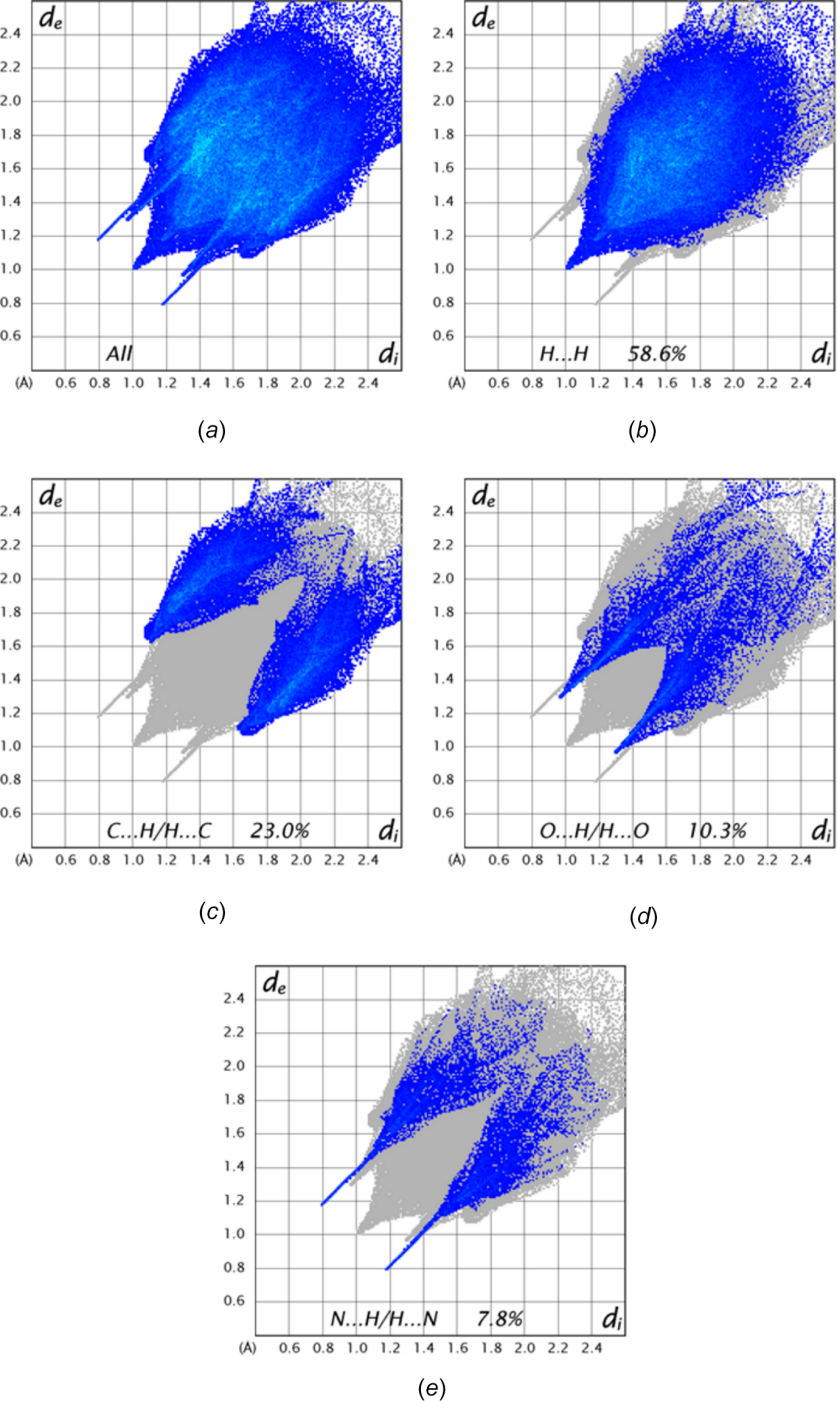
2-D fingerprint plots showing (*a*) all inter­molecular contacts and those delineated into (*b*) H⋯H, (*c*) C⋯H/H⋯C, (*d*) O⋯H/H⋯O and (*e*) N⋯H/H⋯N inter­actions.

A Hirshfeld surface analysis was performed with *CrystalExplorer* (Spackman *et al.*, 2021 ▸) to determine the relative contributions of the several types of inter­molecular inter­actions in the crystal. Details of the process and the inter­pretations of the plots obtained have been published (Tan *et al.*, 2019 ▸). Fig. 6 ▸*a* shows the Hirshfeld surface of the asymmetric unit plotted over *d*_norm_ together with several neighboring mol­ecules, which are hydrogen-bonded to it as described in Section 3. The surface plotted over the curvature function is shown in Fig. 6 ▸*b* from which it is evident that there are no extensive flat regions, which is consistent with the absence of π-stacking inter­actions. Fig. 7 ▸ shows the 2-D fingerprint plots for all inter­molecular contacts (Fig. 7 ▸*a*) as well as those delineated into the four most prominent, specific contacts together with the percent each contributes to the total. More than half come from H⋯H contacts (Fig. 7 ▸*b*), which is consistent with the majority of the hydrogen atoms being part of phenyl and methyl groups and represent the van der Waals contacts. Next in importance are the C⋯H/H⋯C contacts (Fig. 7 ▸*c*), which involve the C—H⋯π (ring) inter­actions tying the layers together (*cf*. Section 3) followed by the O—H/H⋯O and N⋯H/H⋯N contacts (Fig. 7 ▸*d* and *e*, respectively), which represent the C—H⋯O and N—H⋯N hydrogen bonds, respectively. As these involve narrow ranges of donor⋯H and H⋯acceptor distances, they appear as sharp spikes. Other possible contacts contribute very minor amounts.

## 6. Synthesis and crystallization

This compound was synthesized following a method comparable to that described in the literature (Ait Mansour *et al.*, 2024 ▸, 2025 ▸; Ettahiri *et al.*, 2024 ▸).
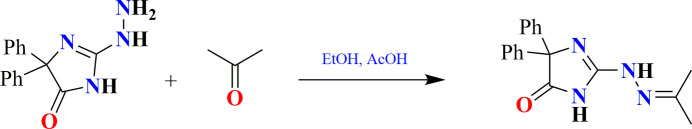


To a solution of 2-hydrazinyl-4,4-diphenyl-1*H*-imidazol-5(4*H*)-one (1.0 g, 3.75 mmol) in ethanol (15 ml), dry acetone (0.3 ml, 4 mmol) was added along with a few drops of acetic acid. The reaction mixture was kept under reflux for 22 h, and then cooled. The precipitated solid was filtered and recrystallized from an ethanol–di­methyl­formamide mixture (9:1), yielding the title compound with a 90% yield, colorless, m.p.479–481 K. FT–IR (ATR, ν, cm^−1^): 3410 (N—H), 3058 (H—C=C), 2918 (CH_3_), 1685 (C=O), 1584, 1552, 1491, 1445 (Ar—C=C). ^1^H NMR (500 MHz, DMSO-*d*_6_): δ_ppm_= 1.97–1.99 (*m*, 6H, 2CH_3_), 7.24–7.48 (*m*, 10H, Ar—H), 9.18 (*s*, 1H, NH—imidazole), 11.48 (*s*, 1H, N—NH). ^13^C NMR (125 MHz, DMSO-*d*_6_); δppm = 18–20 (2CH_3_), 71.90 (C—2Ph), 128.33, 128.79, 129.40, 141.00 (C—Ar); 150.18 (N—N=C), 168.34 (C=N), 180.23 (C=O). HRMS (ESI–MS) (*m*/*z*) calculated for C_18_H_18_N_4_O 307,1481; found 307,15411.

## 7. Refinement

Crystal data, data collection and structure refinement details are summarized in Table 2 ▸. Hydrogen atoms attached to carbon were placed in idealized positions with isotropic displacement parameters tied to those of the attached atoms and included as riding contributions. Those attached to nitro­gen were located in difference maps and refined with a DFIX 0.89 0.01 instruction.

## Supplementary Material

Crystal structure: contains datablock(s) I. DOI: 10.1107/S2056989025000076/zn2041sup1.cif

Structure factors: contains datablock(s) I. DOI: 10.1107/S2056989025000076/zn2041Isup2.hkl

Supporting information file. DOI: 10.1107/S2056989025000076/zn2041Isup3.cml

CCDC reference: 2414562

Additional supporting information:  crystallographic information; 3D view; checkCIF report

## Figures and Tables

**Table 1:** Hydrogen-bond geometry (Å, °) *Cg*5 is the centroid of the C22–C27 benzene ring.

*D*—H⋯*A*	*D*—H	H⋯*A*	*D*⋯*A*	*D*—H⋯*A*
N2—H2⋯O3	0.88 (1)	2.09 (1)	2.952 (2)	165 (2)
N3—H3⋯N8	0.90 (1)	2.22 (1)	3.078 (2)	161 (2)
N5—H5*A*⋯N1	0.88 (1)	2.24 (1)	3.089 (2)	161 (2)
N7—H7*A*⋯N6^i^	0.88 (1)	2.10 (1)	2.964 (2)	172 (2)
C14—H14⋯O1^ii^	0.93	2.55	3.464 (3)	169
C17—H17*B*⋯*Cg*5^iii^	0.96	2.94	3.549 (3)	122
C29—H29⋯O1	0.93	2.43	3.183 (3)	137
C32—H32⋯O2^iv^	0.93	2.40	3.330 (3)	174
C33—H33⋯O2	0.93	2.52	3.131 (3)	124
C35—H35*A*⋯O2^i^	0.96	2.46	3.411 (3)	171

Symmetry codes: (i) 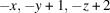; (ii) 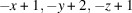; (iii) 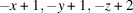; (iv) 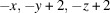.

**Table 2:** Experimental details

Crystal data	
Chemical formula	2C_18_H_18_N_4_O·C_3_H_7_NO
*M*_r_	685.82
Crystal system, space group	Triclinic, *P* 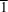
Temperature (K)	293
*a*, *b*, *c* (Å)	9.1009 (3), 11.0914 (6), 18.5952 (9)
α, β, γ (°)	81.381 (4), 83.221 (3), 86.838 (3)
*V* (Å^3^)	1841.52 (15)
*Z*	2
Radiation type	Mo *K*α
μ (mm^−1^)	0.08
Crystal size (mm)	0.34 × 0.26 × 0.17

Data collection	
Diffractometer	SuperNova, Dual, Cu at home/near, Atlas
Absorption correction	Multi-scan (*CrysAlis PRO*; Rigaku OD, 2023 ▸)
*T*_min_, *T*_max_	0.767, 1.00
No. of measured, independent and observed [*I* > 2σ(*I*)] reflections	18303, 8798, 5925
*R*_int_	0.028
(sin θ/λ)_max_ (Å^−1^)	0.697

Refinement	
*R*[*F*^2^ > 2σ(*F*^2^)], *wR*(*F*^2^), *S*	0.060, 0.173, 1.06
No. of reflections	8798
No. of parameters	478
No. of restraints	4
H-atom treatment	H atoms treated by a mixture of independent and constrained refinement
Δρ_max_, Δρ_min_ (e Å^−3^)	0.25, −0.26

Computer programs: *CrysAlis PRO* (Rigaku OD, 2023 ▸), *SHELXS* and *SHELXTL* (Sheldrick, 2008 ▸), *SHELXL2019/1* (Sheldrick, 2015 ▸) and *DIAMOND* (Brandenburg & Putz, 2012 ▸).

## supplementary crystallographic information

### 5,5-Diphenyl-2-[2-(propan-2-ylidene)hydrazin-1-yl]-4,5-dihydro-1H-imidazol-4-one N,N-dimethylformamide hemisolvate Crystal data

**Table:**

2C_18_H_18_N_4_O·C_3_H_7_NO	*Z* = 2
*M**_r_* = 685.82	*F*(000) = 728
Triclinic, *P*1	*D*_x_ = 1.237 Mg m^−^^3^
*a* = 9.1009 (3) Å	Mo *K*α radiation, λ = 0.71073 Å
*b* = 11.0914 (6) Å	Cell parameters from 6730 reflections
*c* = 18.5952 (9) Å	θ = 3.9–28.2°
α = 81.381 (4)°	µ = 0.08 mm^−^^1^
β = 83.221 (3)°	*T* = 293 K
γ = 86.838 (3)°	Block, colourless
*V* = 1841.52 (15) Å^3^	0.34 × 0.26 × 0.17 mm

### 5,5-Diphenyl-2-[2-(propan-2-ylidene)hydrazin-1-yl]-4,5-dihydro-1H-imidazol-4-one N,N-dimethylformamide hemisolvate Data collection

**Table:**

SuperNova, Dual, Cu at home/near, Atlas diffractometer	5925 reflections with *I* > 2σ(*I*)
Detector resolution: 10.5082 pixels mm^-1^	*R*_int_ = 0.028
ω scans	θ_max_ = 29.7°, θ_min_ = 3.3°
Absorption correction: multi-scan (CrysAlisPro; Rigaku OD, 2023)	*h* = −12→12
*T*_min_ = 0.767, *T*_max_ = 1.00	*k* = −15→14
18303 measured reflections	*l* = −24→23
8798 independent reflections	

### 5,5-Diphenyl-2-[2-(propan-2-ylidene)hydrazin-1-yl]-4,5-dihydro-1H-imidazol-4-one N,N-dimethylformamide hemisolvate Refinement

**Table:**

Refinement on *F*^2^	Primary atom site location: structure-invariant direct methods
Least-squares matrix: full	Secondary atom site location: difference Fourier map
*R*[*F*^2^ > 2σ(*F*^2^)] = 0.060	Hydrogen site location: mixed
*wR*(*F*^2^) = 0.173	H atoms treated by a mixture of independent and constrained refinement
*S* = 1.06	*w* = 1/[σ^2^(*F*_o_^2^) + (0.0627*P*)^2^ + 0.7615*P*] where *P* = (*F*_o_^2^ + 2*F*_c_^2^)/3
8798 reflections	(Δ/σ)_max_ = 0.001
478 parameters	Δρ_max_ = 0.25 e Å^−^^3^
4 restraints	Δρ_min_ = −0.26 e Å^−^^3^

### 5,5-Diphenyl-2-[2-(propan-2-ylidene)hydrazin-1-yl]-4,5-dihydro-1H-imidazol-4-one N,N-dimethylformamide hemisolvate Special details

**Table:**

Geometry. All esds (except the esd in the dihedral angle between two l.s. planes) are estimated using the full covariance matrix. The cell esds are taken into account individually in the estimation of esds in distances, angles and torsion angles; correlations between esds in cell parameters are only used when they are defined by crystal symmetry. An approximate (isotropic) treatment of cell esds is used for estimating esds involving l.s. planes.
Refinement. Refinement of F^2^ against ALL reflections. The weighted R-factor wR and goodness of fit S are based on F^2^, conventional R-factors R are based on F, with F set to zero for negative F^2^. The threshold expression of F^2^ > 2sigma(F^2^) is used only for calculating R-factors(gt) etc. and is not relevant to the choice of reflections for refinement. R-factors based on F^2^ are statistically about twice as large as those based on F, and R- factors based on ALL data will be even larger. H-atoms attached to carbon were placed in calculated positions (C—H = 0.93 - 0.96 Å) and were included as riding contributions with isotropic displacement parameters 1.2 - 1.5 times those of the attached atoms. Those attached to nitrogen were placed in locations derived from a difference map and refined with a DFIX 0.89 0.01 instruction.

### 5,5-Diphenyl-2-[2-(propan-2-ylidene)hydrazin-1-yl]-4,5-dihydro-1H-imidazol-4-one N,N-dimethylformamide hemisolvate Fractional atomic coordinates and isotropic or equivalent isotropic displacement parameters (Å^2^)

**Table:**

	*x*	*y*	*z*	*U*_iso_*/*U*_eq_	
O1	0.4540 (2)	0.85849 (14)	0.66174 (9)	0.0675 (5)	
N1	0.42499 (19)	0.66472 (16)	0.72534 (9)	0.0465 (4)	
N2	0.61495 (18)	0.56641 (15)	0.66433 (9)	0.0411 (4)	
H2	0.6793 (19)	0.5062 (15)	0.6559 (12)	0.049*	
N3	0.4718 (2)	0.45755 (16)	0.76305 (10)	0.0469 (4)	
H3	0.3854 (16)	0.456 (2)	0.7917 (11)	0.056*	
N4	0.5561 (2)	0.35323 (16)	0.75034 (10)	0.0478 (4)	
C1	0.6314 (2)	0.69382 (17)	0.63182 (10)	0.0397 (4)	
C2	0.4921 (2)	0.75106 (19)	0.67378 (11)	0.0462 (5)	
C3	0.5036 (2)	0.56028 (18)	0.71804 (10)	0.0398 (4)	
C4	0.7762 (3)	0.73867 (19)	0.65145 (12)	0.0488 (5)	
C5	0.9076 (3)	0.7131 (3)	0.61045 (16)	0.0682 (7)	
H5	0.906542	0.675321	0.569137	0.082*	
C6	1.0413 (3)	0.7436 (3)	0.6305 (2)	0.0881 (9)	
H6	1.129203	0.725437	0.602582	0.106*	
C7	1.0457 (4)	0.7991 (3)	0.6900 (2)	0.0957 (11)	
H7	1.135971	0.819784	0.702671	0.115*	
C8	0.9177 (5)	0.8247 (4)	0.7313 (2)	0.1030 (12)	
H8	0.920500	0.862436	0.772534	0.124*	
C9	0.7818 (4)	0.7945 (3)	0.71222 (16)	0.0811 (9)	
H9	0.694603	0.812384	0.740762	0.097*	
C10	0.6214 (2)	0.71560 (17)	0.54934 (10)	0.0380 (4)	
C11	0.6219 (2)	0.6203 (2)	0.50932 (12)	0.0506 (5)	
H11	0.627681	0.540257	0.532685	0.061*	
C12	0.6138 (3)	0.6433 (2)	0.43450 (13)	0.0607 (6)	
H12	0.614192	0.578411	0.408025	0.073*	
C13	0.6051 (3)	0.7606 (3)	0.39926 (13)	0.0611 (6)	
H13	0.599157	0.775363	0.349097	0.073*	
C14	0.6053 (3)	0.8571 (2)	0.43840 (12)	0.0562 (6)	
H14	0.599980	0.937006	0.414666	0.067*	
C15	0.6134 (2)	0.83423 (19)	0.51344 (11)	0.0465 (5)	
H15	0.613508	0.899166	0.539801	0.056*	
C16	0.5405 (2)	0.2595 (2)	0.79895 (12)	0.0510 (5)	
C17	0.4406 (3)	0.2507 (3)	0.86839 (15)	0.0779 (8)	
H17A	0.407122	0.331127	0.877644	0.117*	
H17B	0.493145	0.211415	0.907886	0.117*	
H17C	0.356955	0.203918	0.864611	0.117*	
C18	0.6338 (3)	0.1493 (2)	0.78539 (18)	0.0827 (9)	
H18A	0.695260	0.166401	0.739721	0.124*	
H18B	0.571276	0.083205	0.783368	0.124*	
H18C	0.694989	0.127075	0.824303	0.124*	
O2	0.21476 (15)	0.77014 (13)	1.04962 (7)	0.0442 (3)	
N5	0.26143 (17)	0.66567 (14)	0.88060 (8)	0.0372 (4)	
H5A	0.287 (2)	0.6600 (19)	0.8338 (6)	0.045*	
N6	0.12404 (16)	0.62615 (14)	0.99037 (8)	0.0363 (3)	
N7	0.09055 (18)	0.51005 (15)	0.89877 (9)	0.0407 (4)	
H7A	0.0235 (19)	0.4673 (17)	0.9278 (10)	0.049*	
N8	0.14707 (18)	0.48358 (15)	0.82887 (9)	0.0425 (4)	
C19	0.29550 (19)	0.76453 (16)	0.91936 (10)	0.0331 (4)	
C20	0.20791 (19)	0.72256 (16)	0.99519 (10)	0.0339 (4)	
C21	0.15679 (19)	0.59687 (16)	0.92148 (10)	0.0341 (4)	
C22	0.46015 (19)	0.76775 (17)	0.92707 (10)	0.0371 (4)	
C23	0.5478 (3)	0.6618 (2)	0.93107 (14)	0.0580 (6)	
H23	0.507930	0.588406	0.926311	0.070*	
C24	0.6963 (3)	0.6652 (3)	0.94226 (17)	0.0785 (8)	
H24	0.754584	0.593397	0.945580	0.094*	
C25	0.7571 (3)	0.7722 (3)	0.94840 (16)	0.0776 (8)	
H25	0.856651	0.773605	0.955071	0.093*	
C26	0.6713 (3)	0.8777 (3)	0.94471 (15)	0.0662 (7)	
H26	0.712254	0.950893	0.949051	0.079*	
C27	0.5226 (2)	0.8752 (2)	0.93446 (12)	0.0485 (5)	
H27	0.464490	0.947033	0.932560	0.058*	
C28	0.23073 (19)	0.88526 (16)	0.88283 (10)	0.0339 (4)	
C29	0.2820 (2)	0.92591 (19)	0.81048 (11)	0.0469 (5)	
H29	0.358759	0.882577	0.786944	0.056*	
C30	0.2207 (3)	1.0296 (2)	0.77290 (12)	0.0588 (6)	
H30	0.255268	1.055053	0.724188	0.071*	
C31	0.1088 (3)	1.0953 (2)	0.80731 (14)	0.0661 (7)	
H31	0.067378	1.165228	0.782081	0.079*	
C32	0.0588 (3)	1.0575 (2)	0.87866 (15)	0.0724 (8)	
H32	−0.016620	1.102227	0.902139	0.087*	
C33	0.1191 (3)	0.9530 (2)	0.91657 (12)	0.0546 (6)	
H33	0.083983	0.928430	0.965298	0.066*	
C34	0.0538 (2)	0.45658 (18)	0.78857 (11)	0.0445 (5)	
C35	−0.1081 (3)	0.4505 (3)	0.80821 (15)	0.0783 (9)	
H35A	−0.128615	0.390377	0.850583	0.117*	
H35B	−0.152954	0.428402	0.768062	0.117*	
H35C	−0.147910	0.528695	0.818739	0.117*	
C36	0.1165 (3)	0.4298 (3)	0.71405 (13)	0.0670 (7)	
H36A	0.212839	0.463130	0.701708	0.101*	
H36B	0.052354	0.466012	0.678608	0.101*	
H36C	0.124278	0.343097	0.714155	0.101*	
O3	0.8365 (2)	0.38834 (19)	0.60869 (13)	0.0860 (6)	
N9	0.8657 (2)	0.1959 (2)	0.58003 (14)	0.0711 (6)	
C37	0.7936 (3)	0.3009 (3)	0.5857 (2)	0.0901 (10)	
H37	0.699437	0.309115	0.570370	0.108*	
C38	1.0119 (4)	0.1762 (4)	0.5999 (3)	0.1186 (14)	
H38A	1.055939	0.253320	0.598302	0.178*	
H38B	1.069647	0.129660	0.566351	0.178*	
H38C	1.008844	0.132129	0.648607	0.178*	
C39	0.8031 (4)	0.0955 (4)	0.5538 (3)	0.1303 (17)	
H39A	0.708134	0.121004	0.537930	0.195*	
H39B	0.791672	0.027932	0.592575	0.195*	
H39C	0.867916	0.071124	0.513504	0.195*	

### 5,5-Diphenyl-2-[2-(propan-2-ylidene)hydrazin-1-yl]-4,5-dihydro-1H-imidazol-4-one N,N-dimethylformamide hemisolvate Atomic displacement parameters (Å^2^)

**Table:**

	*U*^11^	*U*^22^	*U*^33^	*U*^12^	*U*^13^	*U*^23^
O1	0.0965 (13)	0.0415 (9)	0.0531 (10)	0.0172 (8)	0.0198 (9)	0.0007 (7)
N1	0.0549 (10)	0.0415 (9)	0.0389 (9)	0.0023 (8)	0.0067 (7)	−0.0027 (7)
N2	0.0468 (9)	0.0325 (8)	0.0405 (9)	0.0011 (7)	0.0039 (7)	−0.0017 (7)
N3	0.0513 (10)	0.0402 (9)	0.0446 (10)	−0.0041 (8)	0.0049 (8)	0.0016 (8)
N4	0.0541 (10)	0.0386 (9)	0.0477 (10)	−0.0015 (8)	−0.0022 (8)	0.0004 (8)
C1	0.0489 (11)	0.0315 (9)	0.0362 (10)	−0.0007 (8)	0.0014 (8)	−0.0024 (8)
C2	0.0614 (13)	0.0390 (11)	0.0351 (10)	0.0046 (9)	0.0021 (9)	−0.0030 (8)
C3	0.0461 (10)	0.0384 (10)	0.0340 (10)	−0.0040 (8)	−0.0017 (8)	−0.0036 (8)
C4	0.0633 (13)	0.0392 (11)	0.0430 (11)	−0.0093 (10)	−0.0085 (10)	0.0012 (9)
C5	0.0558 (14)	0.0803 (19)	0.0709 (17)	−0.0026 (13)	−0.0116 (12)	−0.0151 (14)
C6	0.0593 (16)	0.100 (2)	0.107 (3)	−0.0079 (16)	−0.0208 (16)	−0.012 (2)
C7	0.090 (2)	0.093 (2)	0.111 (3)	−0.0244 (19)	−0.043 (2)	−0.006 (2)
C8	0.127 (3)	0.114 (3)	0.083 (2)	−0.041 (2)	−0.031 (2)	−0.033 (2)
C9	0.096 (2)	0.092 (2)	0.0634 (17)	−0.0286 (17)	−0.0041 (15)	−0.0300 (16)
C10	0.0372 (9)	0.0397 (10)	0.0354 (10)	−0.0025 (8)	0.0019 (7)	−0.0042 (8)
C11	0.0645 (13)	0.0444 (12)	0.0421 (12)	−0.0096 (10)	0.0032 (10)	−0.0080 (9)
C12	0.0746 (16)	0.0660 (16)	0.0440 (13)	−0.0189 (13)	0.0017 (11)	−0.0169 (11)
C13	0.0631 (14)	0.0823 (18)	0.0360 (11)	−0.0107 (13)	−0.0031 (10)	−0.0014 (12)
C14	0.0598 (13)	0.0575 (14)	0.0449 (12)	0.0012 (11)	−0.0002 (10)	0.0081 (10)
C15	0.0531 (12)	0.0423 (11)	0.0411 (11)	0.0008 (9)	0.0006 (9)	−0.0017 (9)
C16	0.0575 (13)	0.0441 (12)	0.0497 (12)	−0.0074 (10)	−0.0101 (10)	0.0039 (10)
C17	0.094 (2)	0.0692 (17)	0.0593 (16)	−0.0055 (15)	0.0030 (14)	0.0202 (13)
C18	0.086 (2)	0.0507 (15)	0.101 (2)	0.0086 (14)	−0.0019 (17)	0.0114 (15)
O2	0.0528 (8)	0.0464 (8)	0.0347 (7)	−0.0030 (6)	−0.0003 (6)	−0.0128 (6)
N5	0.0445 (8)	0.0362 (8)	0.0302 (8)	−0.0080 (7)	0.0060 (6)	−0.0086 (6)
N6	0.0378 (8)	0.0383 (8)	0.0322 (8)	−0.0036 (7)	0.0021 (6)	−0.0066 (6)
N7	0.0423 (9)	0.0440 (9)	0.0355 (9)	−0.0119 (7)	0.0054 (7)	−0.0087 (7)
N8	0.0467 (9)	0.0449 (9)	0.0367 (9)	−0.0078 (7)	0.0021 (7)	−0.0117 (7)
C19	0.0360 (9)	0.0307 (9)	0.0319 (9)	−0.0022 (7)	0.0021 (7)	−0.0070 (7)
C20	0.0337 (9)	0.0340 (9)	0.0324 (9)	0.0016 (7)	0.0002 (7)	−0.0041 (7)
C21	0.0342 (9)	0.0330 (9)	0.0339 (9)	−0.0015 (7)	0.0006 (7)	−0.0038 (7)
C22	0.0352 (9)	0.0400 (10)	0.0338 (9)	0.0012 (8)	0.0023 (7)	−0.0034 (8)
C23	0.0529 (13)	0.0516 (13)	0.0699 (16)	0.0120 (10)	−0.0079 (11)	−0.0150 (11)
C24	0.0541 (15)	0.091 (2)	0.090 (2)	0.0319 (15)	−0.0153 (14)	−0.0199 (17)
C25	0.0393 (12)	0.114 (3)	0.0791 (19)	0.0008 (15)	−0.0093 (12)	−0.0117 (17)
C26	0.0502 (13)	0.0738 (17)	0.0745 (17)	−0.0187 (13)	−0.0153 (12)	0.0024 (14)
C27	0.0430 (11)	0.0463 (12)	0.0555 (13)	−0.0048 (9)	−0.0081 (9)	−0.0015 (10)
C28	0.0356 (9)	0.0326 (9)	0.0339 (9)	−0.0026 (7)	−0.0034 (7)	−0.0059 (7)
C29	0.0561 (12)	0.0448 (11)	0.0366 (10)	0.0000 (9)	0.0050 (9)	−0.0040 (9)
C30	0.0777 (16)	0.0527 (13)	0.0404 (12)	−0.0002 (12)	−0.0008 (11)	0.0055 (10)
C31	0.0842 (17)	0.0509 (14)	0.0583 (15)	0.0172 (13)	−0.0134 (13)	0.0054 (11)
C32	0.0826 (18)	0.0637 (16)	0.0621 (16)	0.0332 (14)	0.0042 (13)	−0.0036 (13)
C33	0.0609 (13)	0.0544 (13)	0.0414 (11)	0.0184 (11)	0.0061 (10)	−0.0009 (10)
C34	0.0571 (12)	0.0388 (10)	0.0374 (10)	−0.0061 (9)	−0.0070 (9)	−0.0019 (8)
C35	0.0567 (14)	0.124 (3)	0.0563 (15)	−0.0181 (16)	−0.0146 (12)	−0.0090 (16)
C36	0.0878 (18)	0.0712 (17)	0.0458 (13)	−0.0145 (14)	−0.0067 (12)	−0.0169 (12)
O3	0.0787 (13)	0.0637 (12)	0.1172 (18)	0.0015 (10)	0.0023 (12)	−0.0301 (12)
N9	0.0510 (11)	0.0618 (13)	0.1043 (18)	0.0014 (10)	−0.0067 (11)	−0.0275 (12)
C37	0.0521 (15)	0.072 (2)	0.148 (3)	0.0021 (14)	−0.0073 (17)	−0.026 (2)
C38	0.088 (2)	0.118 (3)	0.166 (4)	0.030 (2)	−0.053 (2)	−0.055 (3)
C39	0.101 (3)	0.082 (3)	0.223 (5)	−0.013 (2)	−0.034 (3)	−0.054 (3)

### 5,5-Diphenyl-2-[2-(propan-2-ylidene)hydrazin-1-yl]-4,5-dihydro-1H-imidazol-4-one N,N-dimethylformamide hemisolvate Geometric parameters (Å, º)

**Table:**

O1—C2	1.218 (2)	N7—C21	1.310 (2)
N1—C3	1.343 (3)	N7—N8	1.407 (2)
N1—C2	1.364 (3)	N7—H7A	0.875 (9)
N2—C3	1.333 (2)	N8—C34	1.272 (3)
N2—C1	1.460 (2)	C19—C22	1.525 (2)
N2—H2	0.883 (9)	C19—C28	1.529 (2)
N3—C3	1.333 (2)	C19—C20	1.555 (2)
N3—N4	1.388 (2)	C22—C27	1.381 (3)
N3—H3	0.895 (10)	C22—C23	1.381 (3)
N4—C16	1.274 (3)	C23—C24	1.395 (4)
C1—C10	1.529 (3)	C23—H23	0.9300
C1—C4	1.535 (3)	C24—C25	1.363 (4)
C1—C2	1.564 (3)	C24—H24	0.9300
C4—C9	1.375 (3)	C25—C26	1.368 (4)
C4—C5	1.380 (3)	C25—H25	0.9300
C5—C6	1.387 (4)	C26—C27	1.391 (3)
C5—H5	0.9300	C26—H26	0.9300
C6—C7	1.350 (5)	C27—H27	0.9300
C6—H6	0.9300	C28—C33	1.377 (3)
C7—C8	1.359 (5)	C28—C29	1.387 (3)
C7—H7	0.9300	C29—C30	1.379 (3)
C8—C9	1.398 (4)	C29—H29	0.9300
C8—H8	0.9300	C30—C31	1.371 (3)
C9—H9	0.9300	C30—H30	0.9300
C10—C11	1.380 (3)	C31—C32	1.362 (4)
C10—C15	1.386 (3)	C31—H31	0.9300
C11—C12	1.386 (3)	C32—C33	1.384 (3)
C11—H11	0.9300	C32—H32	0.9300
C12—C13	1.370 (4)	C33—H33	0.9300
C12—H12	0.9300	C34—C35	1.478 (3)
C13—C14	1.382 (4)	C34—C36	1.501 (3)
C13—H13	0.9300	C35—H35A	0.9600
C14—C15	1.391 (3)	C35—H35B	0.9600
C14—H14	0.9300	C35—H35C	0.9600
C15—H15	0.9300	C36—H36A	0.9600
C16—C17	1.483 (3)	C36—H36B	0.9600
C16—C18	1.485 (3)	C36—H36C	0.9600
C17—H17A	0.9600	O3—C37	1.217 (4)
C17—H17B	0.9600	N9—C37	1.316 (4)
C17—H17C	0.9600	N9—C38	1.419 (4)
C18—H18A	0.9600	N9—C39	1.450 (4)
C18—H18B	0.9600	C37—H37	0.9300
C18—H18C	0.9600	C38—H38A	0.9600
O2—C20	1.219 (2)	C38—H38B	0.9600
N5—C21	1.343 (2)	C38—H38C	0.9600
N5—C19	1.465 (2)	C39—H39A	0.9600
N5—H5A	0.884 (9)	C39—H39B	0.9600
N6—C21	1.364 (2)	C39—H39C	0.9600
N6—C20	1.367 (2)		
			
C3—N1—C2	105.43 (16)	N5—C19—C28	109.33 (14)
C3—N2—C1	108.70 (15)	C22—C19—C28	113.47 (14)
C3—N2—H2	125.3 (15)	N5—C19—C20	99.29 (13)
C1—N2—H2	124.9 (15)	C22—C19—C20	110.11 (14)
C3—N3—N4	117.29 (16)	C28—C19—C20	111.17 (14)
C3—N3—H3	117.7 (15)	O2—C20—N6	126.44 (16)
N4—N3—H3	123.2 (15)	O2—C20—C19	124.10 (16)
C16—N4—N3	117.31 (18)	N6—C20—C19	109.45 (15)
N2—C1—C10	113.29 (16)	N7—C21—N5	124.50 (17)
N2—C1—C4	109.06 (16)	N7—C21—N6	122.16 (16)
C10—C1—C4	112.25 (15)	N5—C21—N6	113.33 (16)
N2—C1—C2	98.90 (14)	C27—C22—C23	118.64 (19)
C10—C1—C2	110.66 (16)	C27—C22—C19	120.69 (17)
C4—C1—C2	112.00 (17)	C23—C22—C19	120.56 (18)
O1—C2—N1	126.40 (19)	C22—C23—C24	119.9 (2)
O1—C2—C1	123.29 (18)	C22—C23—H23	120.1
N1—C2—C1	110.28 (16)	C24—C23—H23	120.1
N3—C3—N2	122.63 (18)	C25—C24—C23	120.8 (2)
N3—C3—N1	121.15 (17)	C25—C24—H24	119.6
N2—C3—N1	116.21 (17)	C23—C24—H24	119.6
C9—C4—C5	118.3 (2)	C24—C25—C26	119.8 (2)
C9—C4—C1	122.4 (2)	C24—C25—H25	120.1
C5—C4—C1	119.1 (2)	C26—C25—H25	120.1
C4—C5—C6	120.4 (3)	C25—C26—C27	119.9 (3)
C4—C5—H5	119.8	C25—C26—H26	120.1
C6—C5—H5	119.8	C27—C26—H26	120.1
C7—C6—C5	120.9 (3)	C22—C27—C26	120.9 (2)
C7—C6—H6	119.5	C22—C27—H27	119.5
C5—C6—H6	119.5	C26—C27—H27	119.5
C6—C7—C8	119.7 (3)	C33—C28—C29	118.04 (18)
C6—C7—H7	120.2	C33—C28—C19	123.66 (17)
C8—C7—H7	120.2	C29—C28—C19	118.23 (16)
C7—C8—C9	120.3 (3)	C30—C29—C28	121.00 (19)
C7—C8—H8	119.8	C30—C29—H29	119.5
C9—C8—H8	119.8	C28—C29—H29	119.5
C4—C9—C8	120.4 (3)	C31—C30—C29	120.0 (2)
C4—C9—H9	119.8	C31—C30—H30	120.0
C8—C9—H9	119.8	C29—C30—H30	120.0
C11—C10—C15	118.91 (19)	C32—C31—C30	119.6 (2)
C11—C10—C1	121.87 (17)	C32—C31—H31	120.2
C15—C10—C1	119.22 (17)	C30—C31—H31	120.2
C10—C11—C12	120.4 (2)	C31—C32—C33	120.6 (2)
C10—C11—H11	119.8	C31—C32—H32	119.7
C12—C11—H11	119.8	C33—C32—H32	119.7
C13—C12—C11	120.5 (2)	C28—C33—C32	120.6 (2)
C13—C12—H12	119.7	C28—C33—H33	119.7
C11—C12—H12	119.7	C32—C33—H33	119.7
C12—C13—C14	119.9 (2)	N8—C34—C35	126.2 (2)
C12—C13—H13	120.1	N8—C34—C36	115.8 (2)
C14—C13—H13	120.1	C35—C34—C36	118.0 (2)
C13—C14—C15	119.7 (2)	C34—C35—H35A	109.5
C13—C14—H14	120.2	C34—C35—H35B	109.5
C15—C14—H14	120.2	H35A—C35—H35B	109.5
C10—C15—C14	120.6 (2)	C34—C35—H35C	109.5
C10—C15—H15	119.7	H35A—C35—H35C	109.5
C14—C15—H15	119.7	H35B—C35—H35C	109.5
N4—C16—C17	126.3 (2)	C34—C36—H36A	109.5
N4—C16—C18	116.8 (2)	C34—C36—H36B	109.5
C17—C16—C18	116.9 (2)	H36A—C36—H36B	109.5
C16—C17—H17A	109.5	C34—C36—H36C	109.5
C16—C17—H17B	109.5	H36A—C36—H36C	109.5
H17A—C17—H17B	109.5	H36B—C36—H36C	109.5
C16—C17—H17C	109.5	C37—N9—C38	120.4 (3)
H17A—C17—H17C	109.5	C37—N9—C39	123.1 (3)
H17B—C17—H17C	109.5	C38—N9—C39	116.5 (3)
C16—C18—H18A	109.5	O3—C37—N9	127.3 (3)
C16—C18—H18B	109.5	O3—C37—H37	116.4
H18A—C18—H18B	109.5	N9—C37—H37	116.4
C16—C18—H18C	109.5	N9—C38—H38A	109.5
H18A—C18—H18C	109.5	N9—C38—H38B	109.5
H18B—C18—H18C	109.5	H38A—C38—H38B	109.5
C21—N5—C19	109.78 (14)	N9—C38—H38C	109.5
C21—N5—H5A	122.1 (14)	H38A—C38—H38C	109.5
C19—N5—H5A	126.6 (14)	H38B—C38—H38C	109.5
C21—N6—C20	107.25 (14)	N9—C39—H39A	109.5
C21—N7—N8	114.93 (15)	N9—C39—H39B	109.5
C21—N7—H7A	120.9 (15)	H39A—C39—H39B	109.5
N8—N7—H7A	123.8 (15)	N9—C39—H39C	109.5
C34—N8—N7	116.78 (16)	H39A—C39—H39C	109.5
N5—C19—C22	112.61 (14)	H39B—C39—H39C	109.5
			
C3—N3—N4—C16	170.2 (2)	C21—N5—C19—C22	125.71 (16)
C3—N2—C1—C10	−123.75 (18)	C21—N5—C19—C28	−107.19 (17)
C3—N2—C1—C4	110.46 (18)	C21—N5—C19—C20	9.25 (18)
C3—N2—C1—C2	−6.6 (2)	C21—N6—C20—O2	−175.91 (18)
C3—N1—C2—O1	179.2 (2)	C21—N6—C20—C19	4.26 (19)
C3—N1—C2—C1	−2.3 (2)	N5—C19—C20—O2	172.00 (17)
N2—C1—C2—O1	−176.0 (2)	C22—C19—C20—O2	53.7 (2)
C10—C1—C2—O1	−56.9 (3)	C28—C19—C20—O2	−73.0 (2)
C4—C1—C2—O1	69.2 (3)	N5—C19—C20—N6	−8.17 (18)
N2—C1—C2—N1	5.5 (2)	C22—C19—C20—N6	−126.51 (16)
C10—C1—C2—N1	124.59 (18)	C28—C19—C20—N6	106.86 (17)
C4—C1—C2—N1	−109.3 (2)	N8—N7—C21—N5	6.1 (3)
N4—N3—C3—N2	−2.6 (3)	N8—N7—C21—N6	−174.16 (16)
N4—N3—C3—N1	178.35 (18)	C19—N5—C21—N7	171.89 (17)
C1—N2—C3—N3	−172.76 (19)	C19—N5—C21—N6	−7.9 (2)
C1—N2—C3—N1	6.4 (2)	C20—N6—C21—N7	−177.72 (17)
C2—N1—C3—N3	176.68 (19)	C20—N6—C21—N5	2.0 (2)
C2—N1—C3—N2	−2.4 (2)	N5—C19—C22—C27	154.22 (18)
N2—C1—C4—C9	−92.1 (3)	C28—C19—C22—C27	29.4 (2)
C10—C1—C4—C9	141.5 (2)	C20—C19—C22—C27	−96.0 (2)
C2—C1—C4—C9	16.4 (3)	N5—C19—C22—C23	−29.6 (2)
N2—C1—C4—C5	82.3 (2)	C28—C19—C22—C23	−154.43 (18)
C10—C1—C4—C5	−44.1 (3)	C20—C19—C22—C23	80.2 (2)
C2—C1—C4—C5	−169.3 (2)	C27—C22—C23—C24	−0.1 (3)
C9—C4—C5—C6	0.0 (4)	C19—C22—C23—C24	−176.4 (2)
C1—C4—C5—C6	−174.6 (2)	C22—C23—C24—C25	−0.9 (4)
C4—C5—C6—C7	−0.4 (5)	C23—C24—C25—C26	1.0 (5)
C5—C6—C7—C8	0.6 (6)	C24—C25—C26—C27	−0.2 (4)
C6—C7—C8—C9	−0.4 (6)	C23—C22—C27—C26	0.9 (3)
C5—C4—C9—C8	0.2 (4)	C19—C22—C27—C26	177.2 (2)
C1—C4—C9—C8	174.6 (3)	C25—C26—C27—C22	−0.7 (4)
C7—C8—C9—C4	0.0 (5)	N5—C19—C28—C33	114.8 (2)
N2—C1—C10—C11	−10.2 (3)	C22—C19—C28—C33	−118.6 (2)
C4—C1—C10—C11	113.9 (2)	C20—C19—C28—C33	6.2 (3)
C2—C1—C10—C11	−120.2 (2)	N5—C19—C28—C29	−61.9 (2)
N2—C1—C10—C15	170.62 (17)	C22—C19—C28—C29	64.7 (2)
C4—C1—C10—C15	−65.3 (2)	C20—C19—C28—C29	−170.54 (17)
C2—C1—C10—C15	60.6 (2)	C33—C28—C29—C30	−1.4 (3)
C15—C10—C11—C12	−0.3 (3)	C19—C28—C29—C30	175.4 (2)
C1—C10—C11—C12	−179.5 (2)	C28—C29—C30—C31	0.9 (4)
C10—C11—C12—C13	0.0 (4)	C29—C30—C31—C32	0.0 (4)
C11—C12—C13—C14	0.4 (4)	C30—C31—C32—C33	−0.4 (5)
C12—C13—C14—C15	−0.4 (4)	C29—C28—C33—C32	1.0 (4)
C11—C10—C15—C14	0.3 (3)	C19—C28—C33—C32	−175.7 (2)
C1—C10—C15—C14	179.52 (19)	C31—C32—C33—C28	−0.1 (4)
C13—C14—C15—C10	0.0 (3)	N7—N8—C34—C35	0.0 (3)
N3—N4—C16—C17	−0.1 (4)	N7—N8—C34—C36	179.48 (18)
N3—N4—C16—C18	−178.8 (2)	C38—N9—C37—O3	−1.9 (6)
C21—N7—N8—C34	−143.08 (19)	C39—N9—C37—O3	178.1 (4)

### 5,5-Diphenyl-2-[2-(propan-2-ylidene)hydrazin-1-yl]-4,5-dihydro-1H-imidazol-4-one N,N-dimethylformamide hemisolvate Hydrogen-bond geometry (Å, º)

*Cg*5 is the centroid of the C22–C27 benzene ring.

**Table:**

*D*—H···*A*	*D*—H	H···*A*	*D*···*A*	*D*—H···*A*
N2—H2···O3	0.88 (1)	2.09 (1)	2.952 (2)	165 (2)
N3—H3···N8	0.90 (1)	2.22 (1)	3.078 (2)	161 (2)
N5—H5*A*···N1	0.88 (1)	2.24 (1)	3.089 (2)	161 (2)
N7—H7*A*···N6^i^	0.88 (1)	2.10 (1)	2.964 (2)	172 (2)
C14—H14···O1^ii^	0.93	2.55	3.464 (3)	169
C17—H17*B*···*Cg*5^iii^	0.96	2.94	3.549 (3)	122
C29—H29···O1	0.93	2.43	3.183 (3)	137
C32—H32···O2^iv^	0.93	2.40	3.330 (3)	174
C33—H33···O2	0.93	2.52	3.131 (3)	124
C35—H35*A*···O2^i^	0.96	2.46	3.411 (3)	171

Symmetry codes: (i) −*x*, −*y*+1, −*z*+2; (ii) −*x*+1, −*y*+2, −*z*+1; (iii) −*x*+1, −*y*+1, −*z*+2; (iv) −*x*, −*y*+2, −*z*+2.
